# Reduction of Aflatoxins in Apricot Kernels by Electronic and Manual Color Sorting

**DOI:** 10.3390/toxins8010026

**Published:** 2016-01-19

**Authors:** Rosanna Zivoli, Lucia Gambacorta, Luca Piemontese, Michele Solfrizzo

**Affiliations:** Institute of Sciences of Food Production (ISPA), National Research Council of Italy (CNR), Via Amendola 122/O, Bari 70126, Italy; rosanna.zivoli@ispa.cnr.it (R.Z.); lucia.gambacorta@ispa.cnr.it (L.G.); luca.piemontese@ispa.cnr.it (L.P.)

**Keywords:** aflatoxin, apricot kernels, sorting, processing, occurrence, almonds

## Abstract

The efficacy of color sorting on reducing aflatoxin levels in shelled apricot kernels was assessed. Naturally-contaminated kernels were submitted to an electronic optical sorter or blanched, peeled, and manually sorted to visually identify and sort discolored kernels (dark and spotted) from healthy ones. The samples obtained from the two sorting approaches were ground, homogenized, and analysed by HPLC-FLD for their aflatoxin content. A mass balance approach was used to measure the distribution of aflatoxins in the collected fractions. Aflatoxin B_1_ and B_2_ were identified and quantitated in all collected fractions at levels ranging from 1.7 to 22,451.5 µg/kg of AFB_1_ + AFB_2_, whereas AFG_1_ and AFG_2_ were not detected. Excellent results were obtained by manual sorting of peeled kernels since the removal of discolored kernels (2.6%–19.9% of total peeled kernels) removed 97.3%–99.5% of total aflatoxins. The combination of peeling and visual/manual separation of discolored kernels is a feasible strategy to remove 97%–99% of aflatoxins accumulated in naturally-contaminated samples. Electronic optical sorter gave highly variable results since the amount of AFB_1_ + AFB_2_ measured in rejected fractions (15%–18% of total kernels) ranged from 13% to 59% of total aflatoxins. An improved immunoaffinity-based HPLC-FLD method having low limits of detection for the four aflatoxins (0.01–0.05 µg/kg) was developed and used to monitor the occurrence of aflatoxins in 47 commercial products containing apricot kernels and/or almonds commercialized in Italy. Low aflatoxin levels were found in 38% of the tested samples and ranged from 0.06 to 1.50 μg/kg for AFB_1_ and from 0.06 to 1.79 μg/kg for total aflatoxins.

## 1. Introduction

The main production areas of apricots (*Prunus armeniaca*) are the Mediterranean and Middle East. The top five producers of apricots (fresh fruit) in 2013 were Turkey (811,609 tonnes), Iran (457,308 tonnes), Uzbekistan (430,000 tonnes), Algeria (319,784 tonnes), and Italy (198,290 tonnes) [1] (FAOSTAT). A significant portion of apricots are used to produce apricot stone/pit and its kernel. Apricot kernels are byproducts of the apricot processing industry [2]. In Turkey, 10% of the apricots are used as fresh product, the rest of the product is traditionally stored in sacks with a 20% moisture level after harvesting, sulphuring, drying and stone/pit separation processes [3]. Apricot stones/pits are separated from apricot pulp and processed into shells, mainly used as fuel, and kernels that are exported worldwide, mainly to European countries [4]. Two main varieties of apricot kernels can be easily differentiated, sweet and bitter. Bitter kernels are a good source of amygdaline, which is about 4.5%–6.5% of dry kernels [5]. The oil of bitter kernels (53%) is used in cosmetics and aroma perfume [6] or as a cheaper substitute of bitter almond oil. Apricot kernels can also be of interest as a food or feed ingredient because of their high crude protein content (20%–25% *w*/*w*, dry weight basis) [2]. Sweet apricot kernels can be added to bakery products as whole or ground kernels, as well as consumed as appetizers [7]. Bitter apricot kernels can be used as a substitute of bitter almonds, a more expensive kernel, to produce “persipan” a material used in confectionery and bakery products [5]. The use of persipan as an ingredient of marzipan, made by almond and sugar, at a level >0.5% is considered an adulteration [8]. Moreover, apricot kernels are an important ingredient of the Italian biscuit “amaretti”, whereas the Italian liqueur amaretto is flavoured with extract of these kernels.

Apricot kernels and almonds are at high risk of aflatoxin contamination but they are poorly studied, especially apricot kernels. The first notification of aflatoxins in apricot kernels was published in 1999 in the European Rapid Alert System for Food and Feed (RASFF) network. Between 1999 and 2015 a total of 28 notifications (six alerts, 13 border rejections, nine information) were reported for imported apricot kernels, or derived products produced in Europe, that were found contaminated with high levels of aflatoxins [9]. From 2010 the European maximum levels of aflatoxin B_1_ (AFB_1_) and total aflatoxins (AFs) in apricot kernels intended for further processing (12 µg/kg for AFB_1_ and 15 µg/kg for total AFs) and ready-to-eat (8 µg/kg for AFB_1_ and 10 µg/kg for total AFs) have been aligned to those of Codex Alimentarius after a positive opinion of the European Food Safety Authority (EFSA) [10,11,12,13].

Aflatoxins contamination in maize, peanuts, almonds, pistachios, and Brazil nuts have an extremely uneven distribution [14,15,16,17]. Often the contaminated lots have few kernels contaminated with high levels of aflatoxins, whereas most of kernels have low or no detectable contamination. Sampling plans have been developed to establish the true levels of contamination in lots, but a real correspondence between true levels and results obtained by applying the sampling plans is often not obtained [18,19,20]. The uneven distribution of aflatoxins prompt to the development of strategies of segregation of contaminated kernels from healthy ones, because their removal can drastically reduce aflatoxin contamination of the entire lot. Moreover, it is known that changes of intrinsic characteristics of nuts, like discoloration and staining of skins or kernels, appearance of fluorescent material, changes of size or density, are caused by fungal growth [19]. Some technologies able to detect changes in nut characteristics, such as hand-picking, electronic color sorting (sometimes combined with blanching), size separation, and flotation have been applied to different kind of nuts. Several studies were carried out for peanuts, pistachios, walnuts, and Brazil nuts [21,22,23,24]. Few data are available on almonds [20], whereas apricot kernels have not been studied yet.

The objective of this study was to reduce aflatoxin content in naturally-contaminated apricot kernels by using electronic and manual color sorting as a tool to separate contaminated kernels from healthy ones. The mass balance approach was used to quantitatively determine the distribution of aflatoxins in final and rejected products during sorting processes. The occurrence of aflatoxins was also evaluated on commercial products containing apricot kernels and/or almonds and commercially available in Italy.

## 2. Results and Discussion

### 2.1. Method Performance

The values of limit of detection (LOD) and limit of quantitation (LOQ) obtained for the four aflatoxins in the different matrices considered in this study, and by using the different versions of the analytical method, are reported in Table 1.

Depending on the matrix considered and the amount of matrix equivalent injected into the HPLC system, the values of LOD ranged between 0.03 and 24 μg/kg for AFB_1_, 0.01 and 17.6 for AFB_2_, 0.05 and 3.1 μg/kg for AFG_1_, and 0.02 and 1.26 μg/kg for AFG_2_. The values of LOQ ranged between 0.06 and 48 μg/kg for AFB_1_, 0.02 and 35.2 μg/kg for AFB_2_, 0.1 and 5.40 μg/kg for AFG_1_, and 0.04 and 2.52 μg/kg for AFG_2_. These wide ranges depend of the amount of matrix equivalent, ranging between 1.7 and 150 mg, injected in the HPLC apparatus. In particular, high values of LOD and LOQ were observed for discolored apricot kernels because of the low amount of matrix equivalent injected into the HPLC system and the absence of immuonoaffinity cleanup. On the other hand, low values of LOD and LOQ were not necessary for these naturally contaminated samples which contained high levels of aflatoxins ranging from 1.7 to 22,451.5 µg/kg of AFB_1_ + AFB_2_ (Figure 3 and Figure 4). The values of LOD and LOQ reported in Table 1 were judged acceptable to measure aflatoxin levels occurring in naturally-contaminated apricot kernels and skins, as well as in commercial products containing almonds and/or apricot kernels.

**Table 1:** LOD and LOQ values for determination of AFB_1_, AFB_2_, AFG_1_, and AFG_2_ in apricot kernels, skins, discolored apricot kernels, and commercial products by injecting into HPLC 1.7–150 mg of matrix equivalent.

Matrices	LOD μg/kg				LOQ μg/kg			
	AFB_1_	AFB_2_	AFG_1_	AFG_2_	AFB_1_	AFB_2_	AFG_1_	AFG_2_
Healthy apricot kernels ^a^	0.4	0.08	0.56	0.15	0.8	0.16	1.12	0.30
Discoloured apricot kernels ^b^	24	17.6	-	-	48	35.2	-	-
Apricot kernel skins ^c^	1.40	0.48	3.10	1.26	2.80	0.96	5.40	2.52
Commercial products ^d^	0.03	0.01	0.05	0.02	0.06	0.02	0.1	0.04

^a^ matrix equivalent injected into HPLC apparatus: 25 mg; ^b^ matrix equivalent injected into HPLC apparatus: 1.7 mg; ^c^ matrix equivalent injected into HPLC apparatus: 5 mg; ^d^ matrix equivalent injected into HPLC apparatus: 150 mg.

Results of recovery and repeatability experiments conducted with commercial products are reported in Table 2. Analysis of spiked blank samples demonstrated that the method provides accurate and precise results even at low spiking levels of aflatoxins. In particular, at spiking levels of 0.2 μg/kg of AFB_1_ and 0.5 μg/kg of total AFs, recoveries were 91%–115% and 85%–103%, respectively. At spiking level of 1 μg/kg of AFB_1_ and 2.5 μg/kg of total AFs, recoveries were 92%–98% and 89%–94%, respectively. Repeatability values for AFB_1_ ranged between 2.9% and 6.9% at 0.2 μg/kg and 1.3% and 4.6% at 1 μg/kg. Repeatability values for total AFs ranged between 4.0% and 10.6% at 0.5 μg/kg, and 1.8% and 6.6% at 2.5 μg/kg. Both recovery and repeatability results fulfil the criteria of acceptability of methods for aflatoxin determination in food commodities reported in the EU legislation no. 401/2006 [25].

**Table 2:** Recoveries and within-laboratory repeatability (RSD_r_) for aflatoxins in spiked matrices. Results are mean of three replicates.

Matrices	AFB_1_			Total AF_S_		
	Spiking level (µg/kg)	Recovery (%)	RSD_r_ (%)	Spiking level (µg/kg)	Recovery (%)	RSD_r_ (%)
Almonds	0.2	107	6.9	0.5	100	9.8
	1.0	96	3.4	2.5	94	2.9
Peeled almonds	0.2	102	4.1	0.5	94	10.6
	1.0	94	4.6	2.5	91	6.6
Amaretti	0.2	115	4.1	0.5	103	5.6
	1.0	98	1.3	2.5	94	1.8
Cantucci	0.2	91	5.3	0.5	86	4.0
	1.0	92	2.9	2.5	89	3.5
Almond nougat	0.2	98	5.3	0.5	85	7.1
	1.0	96	3.2	2.5	91	2.5

**Figure 1:**
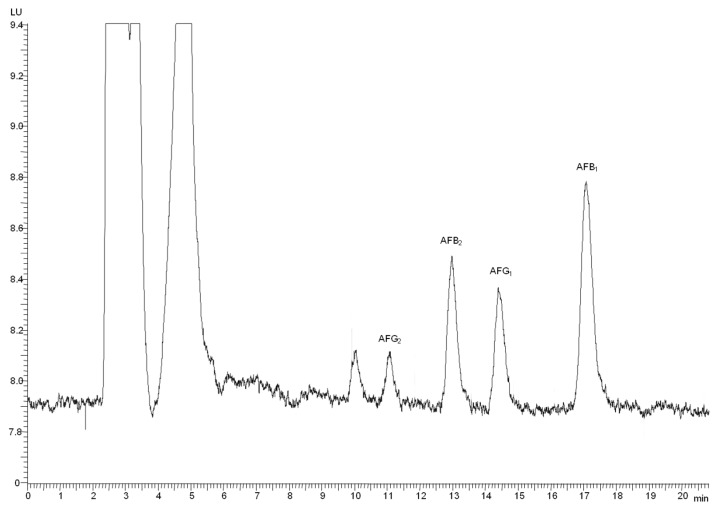
Chromatogram of a commercial sample of salt-roasted almonds with EVO oil naturally contaminated with 0.11 µg/kg AFB_1_, 0.20 µg/kg AFG_1_, 0.01 µg/kg AFB_2_ and AFG_2_.

No interfering peaks at retention times of aflatoxins were visible in the chromatograms of tested sample extracts including those obtained from discolored apricot kernels that were analyzed by HPLC without IMA clean-up. A typical chromatogram of a commercial sample of salt-roasted almonds with EVO oil, naturally-contaminated with the four aflatoxins, is shown in Figure 1.

### 2.2. Electronic Color Sorting (e-Sorting) of Apricot Kernels

**Figure 2:**
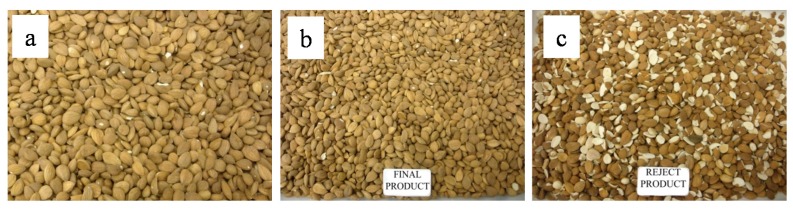
Initial apricot kernels (**a**); final product (**b**) and reject product (**c**) obtained by e-sorting of sample 1.

The 3 × 5 kg samples (1, 2, and 3) of apricot kernels naturally contaminated with aflatoxins were submitted to an e-sorting machine that produced two fractions from each sample, *i.e.*, “final product” and “reject product”. These fractions were weighted, homogenized, and analyzed for aflatoxins. The three fractions of “final product” weighed 4.3 kg, 4.3 kg, and 4.0 kg, whereas the three fractions of “reject product” weighed 0.77 kg, 0.77 kg ,and 0.88 kg. Figure 2 shows the picture of apricot kernels before e-sorting and after e-sorting (“final product” and “reject product”) of sample 1, as an example to explain the e-sorting outcome. From Figure 2 it seems that e-sorting was effective in the segregation of broken kernels from unbroken ones. The amounts of “final product” and “rejected product” were constant for the three samples and ranged between 82% and 85% and 15% and 18% of the initial sample, respectively.

**Figure 3:**
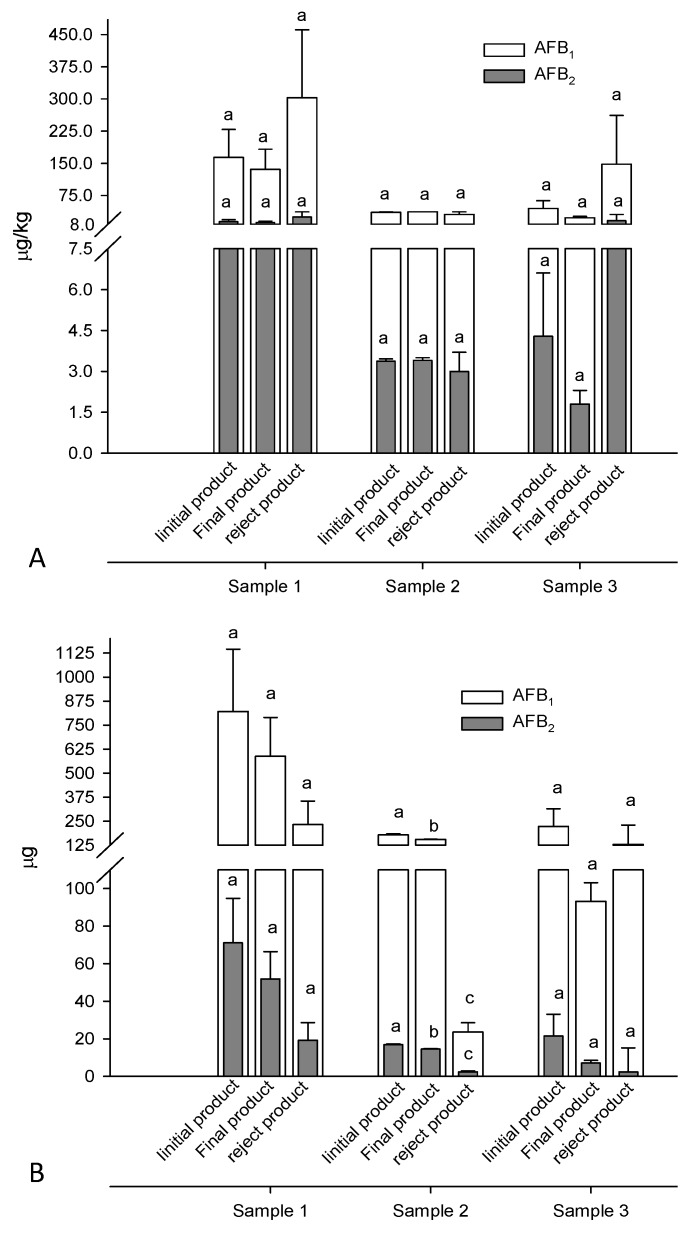
Electronic sorting: (**A**) levels of aflatoxins in initial, final, and reject products; and (**B**) relevant mass balance of aflatoxins. For each sample different letters indicate statistically significant differences among fractions (*p* < 0.05).

All samples of apricot kernels collected from the e-sorter machine were contaminated with AFB_1_ and AFB_2_ whereas AFG_1_ and AFG_2_ were not detected. This means that *A. flavus* was the fungus responsible for aflatoxin accumulation in these samples. The results of AFB_1_ and AFB_2_ levels in the apricot kernels before e-sorting, and in the fractions collected from the e-sorter machine (“final” and “reject”) are shown in Figure 3A. As shown in this figure, comparable levels of AFB_1_ and AFB_2_ were found in the initial apricot kernels, and in the two fractions collected from the e-sorter from each of the three samples. Statistical analysis of these results showed the absence of significant differences within the fractions of samples 1, 2, and 3. This means that the segregation of “reject product” did not result in substantial removal of aflatoxins from the initial samples as the aflatoxin levels measured in the three “reject products” were similar to those measured in the three “final products”. Therefore, there is insufficient evidence to show that aflatoxins accumulate in broken kernels (Figure 2). The mass balance of AFB_1_ and AFB_2_ in these samples is shown in Figure 3B. From this figure it is clear that the amount of aflatoxins measured in the three “reject products” was quite variable with respect to the initial amounts. In particular, it ranged from 13% for sample 2 to 59% for sample 3. This means that after e-sorting the three “final products” still contained 41%–87% of the initial amount of aflatoxins. In other words, the separation of 15%–18% of kernels (“reject product”) removed 13%–59% of total aflatoxins contained in the three samples. Moreover, for samples 1 and 3 no statistical significant difference (*p* > 0.05) was observed for the amount of aflatoxin accumulated in the initial kernels, “final product”, and “reject product”. In conclusion, these results demonstrate that e-sorting was not effective to segregate kernels contaminated with aflatoxins from non-contaminated kernels. The e-sorting was only effective in the segregation of broken kernels from unbroken ones (Figure 2).

E-sorting is the technology most widely used by the processing industries of tree nuts, alone or in combination with other sorting technologies. The e-sorting technology was tested, with variable results, for peanuts, Brazil nuts, pistachios, almonds, and walnuts, but they were not tested for apricot kernels [19,22]. For pistachios, it was reported that the highest AFB_1_ content was measured in discolored pistachios and their segregation from batches produced a total reduction of AFB_1_ that ranged from 94.6% to 98.8% [26]. The electronic and laser color sorters effectively reduced aflatoxins in contaminated lots of almonds [20]. The e-sorting of in-shell Brazil nuts was ineffective in reducing aflatoxins. Therefore the authors suggested, but did not test, to apply the e-sorting to shelled Brazil nuts for a better segregation of contaminated nuts [23].

Electronic color sorting was commonly applied to improve the overall quality of peanuts as well as for reduction of aflatoxins. In a study conducted in 1995 e-sorting produced a 70% of aflatoxin reduction [27]. More recently it was reported that color sorting reduced aflatoxin contamination in shelled peanuts by 29%–38% [21]. In the present study, the application of e-sorting to apricot kernels was ineffective as a stand-alone tool to segregate aflatoxin-contaminated kernels from non-contaminated kernels.

### 2.3. Blanching, Peeling, and Manual Sorting of Apricot kernels

Steaming blanching and peeling, followed by manual color sorting processes, were tested on naturally-contaminated apricot kernels to check the levels and distribution of aflatoxins in skins, healthy, and discolored peeled kernels. The fractions of skins, discolored kernels, and healthy kernels were separately freeze-dried, weighted, homogenized and analyzed for aflatoxin content. The presence of AFB_1_ and AFB_2_, at various levels, was confirmed also in these samples. The weight percentage of skins ranged between 5.5% and 6.1% for the 3 × 500 g kernel samples. Skins contained 4.8%–5.7% of total aflatoxins present in the initial kernels; therefore, skin removal did not reduce the aflatoxin content in peeled kernels. Our observation is consistent with the results obtained for almonds artificially contaminated with AFB_1_ and AFB_2_ and submitted to blanching and peeling [28].

**Figure 4:**
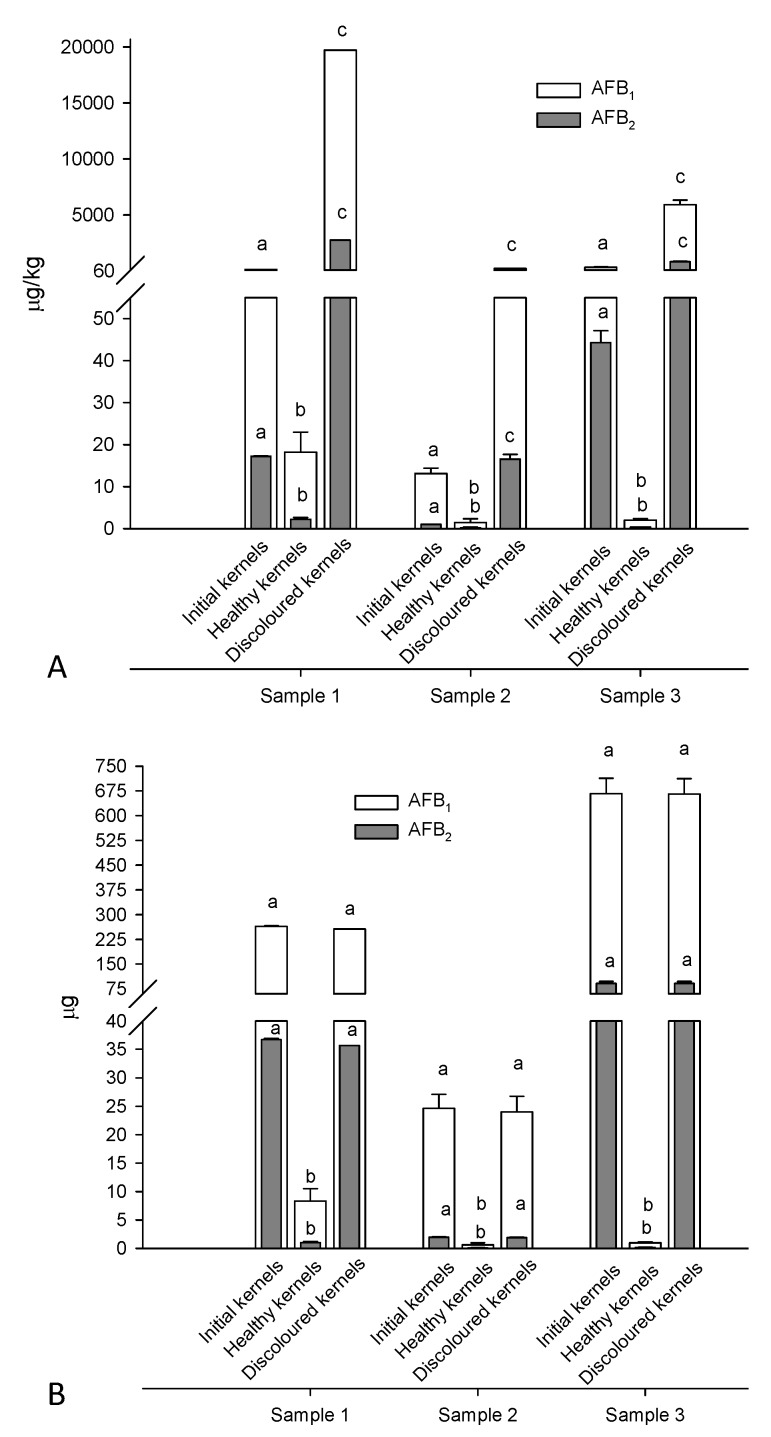
Manual sorting of naturally-contaminated apricot kernels: (**A**) levels of aflatoxins in initial, healthy and discolored kernels; and (**B**) relevant mass balance of aflatoxins. For each sample different letters indicate statistically significant differences among fractions (*p* < 0.05).

Aflatoxin levels in peeled apricot kernels before and after manual separation into healthy and discolored ones are reported in Figure 4A. The highest levels of aflatoxins were found in discolored kernels, followed by initial and healthy ones. The manual separation of discolored kernels was, therefore, quite effective in removing almost all aflatoxins present in the initial samples as demonstrated by their mass balance (Figure 4B). In particular, 97.3%–99.5% of the total aflatoxins present in the initial peeled kernels accumulated in the discolored kernels and only 0.5%–2.7% remained in the healthy ones. The weight percentage of discolored kernels ranged between 2.6% and 19.9% for the 3 × 500 g kernel samples. Therefore, the segregation of 2.6%–19.9% (mean 9.5%) of kernels removed 97.3%–99.5% of total aflatoxins (Figure 4B).

**Figure 5:**
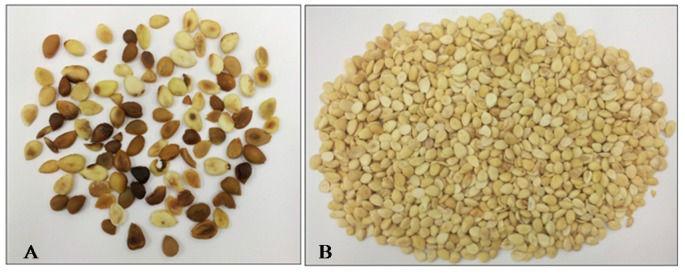
(**A**) discolored apricot kernel (6.2% of total peeled kernels) contained 99.5% of total aflatoxins contained in peeled kernels (sample 3); and (**B**) healthy apricot kernels (93.8% of total peeled kernels) contained 0.5% of total aflatoxins contained in peeled kernels (sample 3).

The picture of discolored (A) and healthy (B) kernels obtained from blanching and manual sorting of 0.5 kg of apricot kernels (sample 3) is shown in Figure 5. It was easy to visually identify and segregate discolored kernels having different degrees of discoloration that ranged from a single spot to the entire kernel. Unpeeled discolored kernels could not be distinguished from unpeeled healthy kernels because skins of both types of kernels have the same brown color. This could be the reason why the e-sorting machine could not identify and segregate discolored kernels from healthy ones (see Figure 3 and Figure 4). The combination of peeling (blanching) and e-sorting could be, therefore, the optimal procedure to automatically separate contaminated kernels from uncontaminated kernels, providing that the machine is able to identify and separate both kernels uniformly discolored and kernels having a single spot. This approach was successfully used for peanuts with aflatoxin reduction between 83% and 91% [14,27]. In developed countries, hand picking is still used in certain cases for peanuts, in spite of its high cost, to achieve a better removal of contaminated material [18,29].

### 2.4. Occurrence of Aflatoxins in Commercial Products Containing Apricot Kernels and/or Almonds

The improved HPLC-FLD method having low values of LOD and LOQ was used herein to determine the occurrence of aflatoxins in commercial products containing apricot kernels and/or almonds, the two tree nuts considered in this study. In Table 3 are reported the results of 47 commercial products of which 39 were almonds and derived products and eight were apricot kernel flour-based products. Overall, 18 samples (38%) were found contaminated with aflatoxins at levels ranging from 0.06 to 1.50 μg/kg of AFB_1_ and 0.06 to 1.79 μg/kg of total AFs. Although a high percentage of samples were contaminated, the measured levels of AFB_1_ and total AFs were below the limits set by the European Legislation for these products *i.e.*, 8 μg/kg AFB_1_ and 10 μg/kg total AFs [12] (EU 165/2010). Among the 39 almonds and derived products, 11 samples (28%) were contaminated with a mean level of AFB_1_ and total AFs of 0.07 and 0.11 µg/kg, respectively. The six samples of nougat were all found negative. The absence of aflatoxins in these samples may be related to the type of processing used for the preparation of this almond-derived product which involves caramelization of sugar. We have previously demonstrated that the caramelization of sugar spiked with aflatoxins reduce aflatoxin levels up to 82% and similar results were obtained when contaminated almonds were processed into nougat at laboratory scale [28].

Among the eight apricot kernel-derived products (amaretti), seven samples (88%) were contaminated with a mean level of AFB_1_ and total AFs of 0.63 and 0.81 µg/kg, respectively. The percentage in weight of almonds in commercial products considered in this survey ranged from 7% to 100% of the final product. The percentage in weight of apricot kernels ranged from 16% to 40% for amaretti. However, no correlation was found between the percentage of almonds or apricot kernels and the aflatoxin contamination, which confirms the uneven distribution of aflatoxin contamination. On the other hand, the percentage of positive samples and mean contamination levels of apricot kernel-derived products are consistently higher as compared to almonds and almond-derived products (Table 3). In addition to the higher percentage of positive samples, the mean levels of AFB_1_ and total AFs in apricot kernel derived products were 7–9 times higher the levels found in almonds and almond-derived products. From these data, it can be concluded that the risk of aflatoxin contamination seems to be higher for apricot kernel derived products as compared to almond-derived products. The occurrence of aflatoxins in apricot kernels and derived products was poorly investigated in the past, as demonstrated by the limited number of papers in the scientific literature [30,31].

**Table 3:** Occurrence of aflatoxins in commercial products containing almonds and/or apricot kernels and commercially available in Italy.

Commercial Product	% of Almonds or Apricot Kernels	Origin	AFB_1_ (µg/kg)	Total AFs (µg/kg)
Almonds	100%	Italy	0.06	0.06
Almonds	100%	California, US	n.d.	n.d.
Almonds	100%	California, US	n.d.	n.d.
Almonds	100%	Italy ^b^	n.d.	n.d.
Almonds	100%	Italy	n.d.	n.d.
Almonds	100%	Italy ^b^	n.d.	n.d.
Almonds	100%	Italy	n.d.	n.d.
Peeled almonds	100%	Italy	0.06	0.07
Peeled almonds	100%	California, US	0.06	0.06
Peeled almonds	100%	Italy ^b^	0.06	0.06
Peeled almonds	100%	Italy	0.06	0.06
Peeled almonds	100%	California, US	n.d.	n.d.
Peeled almonds	100%	California, US	n.d.	n.d.
Almond flour	100%	Italy	0.06	0.06
Chopped almonds	100%	Italy ^b^	0.06	0.06
Bakery product (4% almonds) ^a^	100%	Italy ^b^	0.06	0.16
Bakery product (4% almonds) ^a^	100%	Italy ^b^	n.d.	n.d.
Bakery product (3% chopped peeled almonds) ^a^	100%	Italy ^b^	n.d.	n.d.
Bakery product (3% almonds) ^a^	100%	Italy ^b^	n.d.	n.d.
Bakery product (3% peeled almonds) ^a^	100%	Italy ^b^	n.d.	n.d.
Bakery product (4% peeled almonds) ^a^	100%	Italy ^b^	n.d.	n.d.
Bakery product (4% peeled almonds) ^a^	100%	Italy ^b^	n.d.	n.d.
Bakery pastries	7% Almonds	Italy ^b^	n.d.	n.d.
Mixed nuts snack (almonds, peanuts, sultana grapes, cranberry)	25% Almonds	California, US	n.d.	n.d.
Salt roasted almonds with EVO oil	100%	Italy	0.11	0.33
Salt roasted almonds	100%	Italy ^b^	n.d.	n.d.
Smoked almonds	100%	Italy ^b^	n.d.	n.d.
Sugared almonds	100%	Italy	n.d.	n.d.
Almonds soft nougat	50% Almonds	Italy ^b^	n.d.	n.d.
Almonds nougat	45% Almonds	Italy ^b^	n.d.	n.d.
Almonds nougat	45% Almonds	Italy ^b^	n.d.	n.d.
Almonds soft nougat	40% Almonds	Italy ^b^	n.d.	n.d.
Almonds nougat	36% Almonds	Italy ^b^	n.d.	n.d.
Almonds soft nougat	30% Almonds	Italy ^b^	n.d.	n.d.
Cantucci	22% Almonds	Italy ^b^	0.06	0.07
Cantucci	22% Almonds	Italy ^b^	0.06	0.06
Cantucci	22% Almonds	Italy ^b^	n.d.	n.d.
Cantucci	20% Almonds	Italy ^b^	n.d.	n.d.
Cantucci	19% Almonds	Italy ^b^	n.d.	n.d.
Mean of positive samples	-	-	0.07	0.11
Amaretti	38% Apricot kernels, 4% Almonds, 4% Pistachios	Italy ^b^	1.30	1.79
Amaretti	40% Apricot kernels, 5% Almonds	Italy ^b^	1.50	1.56
Amaretti	20% Apricot kernels	Italy ^b^	0.55	0.99
Amaretti	20% Apricot kernels	Italy ^b^	0.53	0.56
Amaretti	19% Apricot kernels	Italy ^b^	0.40	0.41
Amaretti	16% Apricot kernels, Sweet and bitter almonds	Italy ^b^	0.08	0.30
Amaretti	20% Apricot kernels	Italy ^b^	0.06	0.06
Amaretti	20% Apricot kernels	Italy ^b^	n.d.	n.d.
Mean of positive samples	-	-	0.63	0.81

n.d. = not detected; ^a^ relative percentage of almonds in the whole product; ^b^ manufacturing country.

## 3. Experimental Section

### 3.1. Samples

*Apricot kernels.* Samples of apricot kernels (20 kg) originating from Turkey were provided by a local importer (Bari, Italy) and used for electronic and manual color sorting experiments.

*Commercial products.* Forty-seven commercial products containing apricot kernels and/or almonds were analysed for aflatoxins content (AFB_1_, AFB_2_, AFG_1_, and AFG_2_). In particular, seven samples of almonds, six samples of peeled almonds, two samples of roasted almonds, one sample of bakery pastries, five samples of Cantucci pastries, eight samples of amaretti mini biscuits, seven bakery products containing peeled almonds (four) or almonds (three); six samples of almond nougat, and five other products (almond flour, chopped almonds, smoked almonds, sugared almonds praline, and mixed nuts snack). The seven bakery products (“panettone” and “colomba”) containing embedded whole almonds were crumbled to pick up the almonds that were analyzed separately. Each sample was finely ground by blending and analysed for aflatoxins.

### 3.2. Electronic Color Sorting of Apricot Kernels

The e-sorting experiments were performed in triplicate by using an optical sorter machine (Bühler Z+) (Bühler, Brescia, Italy). In particular, 15 kg of apricot kernels naturally contaminated with aflatoxins were manually mixed and divided in 3 × 5 kg aliquots that were singularly submitted to e-sorting. For each of the three aliquots the machine separated the kernels in two fractions called “final” and “reject”. Final and reject fractions of the 3 × 5 kg aliquots were singularly collected, weighted, slurried, or homogenized and analyzed for their aflatoxin content. With this approach, the initial aflatoxin concentration of the 15 kg of kernels was deduced from the results of the analysis of the whole “final” and “reject” fractions. Fractions weighting ≥4 kg were slurried (1:1.5 with water) using the slurry mixer Silverson (Silverson Machines Ltd., Waterside, Chesham, UK), whereas the fractions that could not be slurried, because of low amount (<1 kg), were homogenized by blending (DitoSama, Model F23200, Aubusson, France).

### 3.3. Blanching, Peeling, and Manual Sorting of Apricot Kernels

Steam blanching was carried out in a steamer (model HD 9140, Philips Electronics, Eindhoven, The Netherlands) for 40 min on 3 × 500 g of apricot kernels naturally contaminated with aflatoxins. After steam blanching, apricot kernels were manually peeled to separate peeled kernels from skins. Peeled kernels were further manually sorted to separate discolored kernels (brown/dark and spotted ones) from healthy ones. Samples of skins and peeled kernels (discolored and healthy) were separately freeze-dried, weighed, homogenized, and analyzed for their aflatoxin content for a total of nine samples. Each sample was analyzed in triplicate for a total of 27 analyses. The analytical method for aflatoxin determination is describe below.

### 3.4. Determination of AFB_1_, AFB_2_, AFG_1_ and AFG_2_

The HPLC-FLD method previously described [28] was used herein with some changes depending of the nature of samples analysed.

*Aflatoxin determination in samples of apricot kernels and skins derived from e-sorting and manual sorting.* Aflatoxins were extracted from 10 g of dry sample by sonication for 30 min with 100 mL of acetone:water (85:15 *v/v*) in 250 mL Pyrex screw-capped glass flasks. Extracts were filtered on filter paper (No. 4, Whatman, Maidstone, UK) and 5 mL of filtered extract were diluted with 75 mL of ultrapure water and filtered through glass microfilter (GF/A, Whatman, Maidstone, UK). A 40 mL volume of filtered diluted extract (equivalent to 0.25 g of matrix) was passed through the immunoaffinity column (IAC) AflaTest (Vicam, Milford, MA, USA) at the flow rate of 1–2 drops/second. Then the column was washed with 2 × 10 mL of ultrapure water that was discarded. Aflatoxins were eluted from the column by passing 3 × 0.5 mL of methanol. The eluates were collected and diluted with ultrapure water up to 5 mL in a volumetric flask. Volume of 100–500 μL (equivalent to 5–25 mg of matrix) were injected in the HPLC-FLD apparatus.

*Aflatoxin determination in discolored apricot kernels.* These samples were supposed to contain high levels of aflatoxins therefore, the sample extracts were diluted and directly analysed by HPLC-FLD without using the immunoaffinity clean-up. In particular, 10 g were extracted with acetone:water (85:15 *v*/*v*), and 0.5 mL of the filtered extract was diluted with 30 mL of MeCN:H_2_O (30:70 *v*/*v*), vortexed, filtered through a 0.45 μm PTFE filter (Sartorius Stedim Italy SpA, Bagno a Ripoli, Firenze, Italy) and injected into the HPLC apparatus (the injection volume was 100 μL equivalent to 1.7 mg of matrix).

*Analysis of commercial products.* To obtain low limits of detection the method was modified to get a more concentrated final extracts for HPLC analysis. Briefly, 10 g of dry ground sample was weighted in a 250 mL Pyrex screw-capped glass flask and extracted by sonication for 30 min with 50 mL of extraction mixture of acetone:water (85:15 *v*/*v*). After filtration on filter paper (No. 4, Whatman, Maidstone, UK) 10 mL of filtered extract were diluted with 140 mL of ultrapure water and filtered with a GF/A glass microfiber (Whatman, Maidstone, UK). The immunoaffinity clean-up was performed by passing 113 mL of filtered diluted extract (equivalent to 1.5 g of matrix) through the IAC column at the flow rate of 1–2 drops/min. After washing the IAC column with 2 × 10 mL of ultrapure water, aflatoxins were eluted with 0.75 mL methanol, and after 1 min eluted again with 0.5 mL methanol. The collected eluates were diluted with ultrapure water up to 5 mL in a volumetric flask and analyzed by HPLC-FLD. Injection volumes were 100–500 μL equivalent to 30–150 mg of matrix.

### 3.5. Chemicals and Reagents

Methanol (MeOH), acetonitrile (MeCN) and acetone were purchased from Sigma Aldrich (Milan, Italy). Ultrapure water was produced with a Milli-Q system (Millipore, Bedford, MA, USA). AflaTest WB wide bore affinity columns were purchased from Vicam L.P. (Watertown, MA, USA). The mixed aflatoxins standard solution (purity 99% ± 1%), prepared in acetonitrile and containing 2.00 µg/mL AFB_1_, 2.02 µg/mL AFG_1_, 0.50 µg/mL AFB_2_ and 0.50 µg/mL AFG_2_, was purchased from Romer Labs Diagnostic (Tulln, Austria). This solution was used to prepare calibration solutions for HPLC-FLD determinations and recovery experiments.

### 3.6. HPLC-FLD Apparatus and Conditions

The HPLC analyses of aflatoxins were performed by using a mixture of acetonitrile: water (30:70) as mobile phase. A Perkin Elmer Series 200 binary pump was used at a flow rate of 0.8 mL/min (the run time was 20 min). Sample extracts were injected into the HPLC apparatus via a Rheodyne 7125 manual injection valve equipped with a 500 μL sample loop. The apparatus was equipped with a chromatographic data handling software for Microsoft Windows XP (PerkinElmer TotalChrom Workstation version 6.3.1) (Perkin Elmer, Waltham, MA, USA). The separation of aflatoxins was obtained with a Luna^®^ analytical column PFP (2) (pentafluorophenyl propyl), 150 × 4.6 mm i.d., 3 μm, 100 Å (Phenomenex, Torrance, CA, USA), preceded by a security guard cartridge (4 × 3 mm, 5 μm) (Phenomenex). Aflatoxin detection was carried out using a Jasco FP-2020 plus fluorescence detector set at 365 nm (λ_ex_) and 435 nm (λ_em_). A photochemical post-column derivatisation (UVE™ system, LCTech, Dorfen, Germany) was used to enhance the fluorescence of AFB_1_ and AFG_1_.

### 3.7. Calibration Curves

A mixed aflatoxins stock solution was prepared by diluting 500 μL of commercial standard solution (2 µg/mL AFB_1_ and AFG_1_, 0.5 µg/mL AFB_2_ and AFG_2_) to 10 mL with acetonitrile in a volumetric flask. The five HPLC calibration solutions of combined aflatoxins (AFB_1_ + AFB_2_ + AFG_1_ + AFG_2_) were prepared by diluting appropriate volumes of the stock solution with MeOH:H_2_O (40:60 *v*/*v*). The toxin concentration ranges of the five calibration solutions were 0.10–3.64 ng/mL for AFB_1_ and AFG_1_, 0.025–0.90 ng/mL for AFB_2_ and AFG_2_.

### 3.8. Recovery Experiments

The performances of the improved HPLC/FLD method were assessed by performing recovery and repeatability experiments. Blank samples of almonds, peeled almonds, amaretti, cantucci, and almond nougat were spiked with mixtures of the four aflatoxins and analyzed in triplicate. Two spiking levels of aflatoxins were tested (0.2 μg/kg and 1 μg/kg for AFB_1_; 0.5 μg/kg and 2.5 μg/kg for total AFs). In particular, for recovery experiments at spiking levels of 0.2 μg/kg of AFB_1_ and 0.5 μg/kg for total AFs, 3 × 10 g of samples were spiked with 3 × 20 µL of the spiking solution. For experiments at spiking levels of 1 μg/kg of AFB_1_ and 2.5 μg/kg of total AFs, 3 × 100 μL of the same spiking solution were added to 3 × 10 g of samples. Spiked samples were left overnight in the dark at room temperature to allow solvent evaporation. The values of LOD and LOQ were calculated as signal-to-noise ratio of three and six, respectively.

### 3.9. Statistical Analysis

Mean and standard deviation (SD) of data were calculated with a SigmaPlot for Windows version 12.0 statistical software package (Sistat, Software, Inc., Chicago, IL, USA). SigmaPlot was also used to perform one-way analysis of variance (ANOVA) followed by a Tukey pairwise multiple comparison test, and a least significant difference (LSD) test at 95% confidence levels (*p* = 0.05) to identify significant differences among groups.

## 4. Conclusions

This study, conducted with naturally contaminated apricot kernels, has demonstrated for the first time that the combination of peeling and visual/manual separation of discolored kernels is a feasible strategy to remove up to 99% of aflatoxins in this food commodity. Therefore, the existing sorting machines should be used to segregate peeled apricot kernels instead of unpeeled kernels.

The occurrence of aflatoxins in almond and apricot kernel derived products commercially available in Italy has been demonstrated for 38% of tested samples. However, the measured levels of aflatoxins in positive samples were all below the maximum limits established by EU regulation for these products. This is the first study that has analysed pastries containing apricot kernels (amaretti) and has demonstrated that almost all tested products were contaminated at levels 7–9 times higher than those found in almond based products.
